# A Fibreoptic endoscopic study of upper gastrointestinal bleeding at Bugando Medical Centre in northwestern Tanzania: A retrospective review of 240 cases

**DOI:** 10.1186/1756-0500-5-200

**Published:** 2012-07-03

**Authors:** Hyasinta Jaka, Mheta Koy, Anthony Liwa, Rodrick Kabangila, Mariam Mirambo, Wolfgang Scheppach, Eliasa Mkongo, Mabula D Mchembe, Phillipo L Chalya

**Affiliations:** 1 Department of Internal Medicine, Bugando Medical Centre, Mwanza, Tanzania; 2 Department of Pharmacology, Catholic University of Health and Allied Sciences-Bugando, Mwanza, Tanzania; 3 Department of Microbiology and Immunology, Catholic University of Health and Allied Sciences-Bugando, Mwanza, Tanzania; 4 Department of Gastroenterologie and Rheumatologie, Stiftung Juliusspital, Juliuspromenade, Wurzburg, Germany; 5 Department of Surgery, Amana Hospital and Hubert Kairuki Memorial University, Dar Es Salaam, Tanzania; 6 Department of Surgery, Muhimbili University of Health and Allied Sciences, Dar Es Salaam, Tanzania; 7 Department of Surgery, Catholic University of Health and Allied Sciences-Bugando, Mwanza, Tanzania

**Keywords:** Fibreoptic endoscopy, Upper gastrointestinal bleeding, Aetiological spectrum, Clinical profile, Management, Clinical outcome, Tanzania

## Abstract

**Background:**

Upper gastrointestinal (GI) bleeding is recognized as a common and potentially life-threatening abdominal emergency that needs a prompt assessment and aggressive emergency treatment. A retrospective study was undertaken at Bugando Medical Centre in northwestern Tanzania between March 2010 and September 2011 to describe our own experiences with fibreoptic upper GI endoscopy in the management of patients with upper gastrointestinal bleeding in our setting and compare our results with those from other centers in the world.

**Findings:**

A total of 240 patients representing 18.7% of all patients (i.e. 1292) who had fibreoptic upper GI endoscopy during the study period were studied. Males outnumbered female by a ratio of 2.1:1. Their median age was 37 years and most of patients (60.0%) were aged 40 years and below. The vast majority of the patients (80.4%) presented with haematemesis alone followed by malaena alone in 9.2% of cases. The use of non-steroidal anti-inflammatory drugs, alcohol and smoking prior to the onset of bleeding was recorded in 7.9%, 51.7% and 38.3% of cases respectively. Previous history of peptic ulcer disease was reported in 22(9.2%) patients. Nine (3.8%) patients were HIV positive. The source of bleeding was accurately identified in 97.7% of patients. Diagnostic accuracy was greater within the first 24 h of the bleeding onset, and in the presence of haematemesis. Oesophageal varices were the most frequent cause of upper GI bleeding (51.3%) followed by peptic ulcers in 25.0% of cases. The majority of patients (60.8%) were treated conservatively. Endoscopic and surgical treatments were performed in 30.8% and 5.8% of cases respectively. 140 (58.3%) patients received blood transfusion. The median length of hospitalization was 8 days and it was significantly longer in patients who underwent surgical treatment and those with higher Rockall scores (P < 0.001). Rebleeding was reported in 3.3% of the patients. The overall mortality rate of 11.7% was significantly higher in patients with variceal bleeding, shock, hepatic decompensation, HIV infection, comorbidities, malignancy, age > 60 years and in patients with higher Rockall scores and those who underwent surgery (P < 0.001).

**Conclusion:**

Oesophageal varices are the commonest cause of upper gastrointestinal bleeding in our environment and it is associated with high morbidity and mortality. The diagnostic accuracy of fibreoptic endoscopy was related to the time interval between the onset of bleeding and endoscopy. Therefore, it is recommended that early endoscopy should be performed within 24 h of the onset of bleeding.

## Background

Upper gastrointestinal (GI) bleeding, defined as bleeding derived from a source proximal to the ligament of Treitz, is a common and potentially life-threatening abdominal emergency that remains a common cause of morbidity and mortality worldwide [1,2]. Bleeding from the upper GI tract is approximately 4 times as common as bleeding from the lower GI tract [3]. The disease spectrum has a wide range of clinical severity, ranging from insignificant bleeds to catastrophic exsanguinating hemorrhage [4]. Approximately 80%–85% of upper GI bleeding stops spontaneously and supportive therapy only is required [1,5]. In the remaining 15%–20% of cases bleeding continues or recurrent bleeding develops, and these patients constitute the high-risk group with substantially increased morbidity and mortality [6]. Upper GI bleeding is responsible for more than 300,000 hospital admissions annually in the United States, with a mortality rate of 7% to 10% [7]. In the United Kingdom, the incidence of upper GI bleeding is 50-190/10,000/year and is highest in areas of social deprivation [2,8].The incidence of upper GI bleeding is 2-fold greater in males than in females, in all age groups; however, the death rate is similar in both sexes [8]. In Bugando Medical Centre, upper GI bleeding is one of the most common causes of admissions to the medical and surgical wards and contributes significantly to high morbidity and mortality.

The aetiology and outcome of upper GI bleeding varies significantly in different geographic regions depending on the demographic and socioeconomic characteristics of the local population [9]. Causes of upper GI bleeding have been classified to variceal (e.g. esophageal and gastric varices) and non variceal (e.g. peptic ulcer, erosive gastroduodenitis, reflux esophagitis, tumors, vascular ectasia etc.) [9,10]. Previous studies in northern Tanzania showed that the leading causes of upper GI bleeding were oesophageal varices [11,12], which is at variance with other reports in developed countries which reported peptic ulcers as the most common cause of upper GI bleeding [13-15].

Fiberoptic upper GI endoscopy has recently been recognized as the standard investigation of choice for patients with upper GI bleeding since it plays a pivotal role in the diagnosis and therapy of these patients, reducing mortality, rebleeding, requirement for transfusion, the need for surgery, hospital stay and health care costs [11]. Timely endoscopy plays a central role in the modern management of acute upper GI bleeding with the value of endoscopic therapy for bleeding from upper GI being well established [13,16]. In Tanzania and many developing countries, upper GI endoscopy services are not readily available or affordable for most patients. This has limited the gathering of precise data on the aetiology of upper GI bleeding as most patients are treated without any endoscopic evaluation to assess the aetiology and response to treatment [11,12].

Despite recent development of new therapeutic tools such as the proton pump inhibitors, endoscopic interventions and surgical approaches, the overall clinical outcome of patients with UGIB has not changed significantly during the past decade and mortality rate remains around 10% in most studies reported in the literature [7,17]. Several scoring systems have been developed to predict the risk of adverse clinical outcome in patients with upper gastrointestinal bleeding [4,7]. Rockall *et al.*[4] developed a risk-scoring system involving clinical and endoscopic criteria to predict the risk of rebleeding and mortality in patients with upper GI bleeding. The Rockall scoring system is feasible, accurate, effective system for predicting outcome in patients with upper GI bleeding.

Our centre introduced the endoscopy services about five years ago and we report our experiences with fiberoptic upper GI endoscopy in the diagnosis and treatment of upper gastrointestinal bleeding in patients who were referred to our centre for endoscopy. The study outlines the aetiology, clinical presentation and treatment outcome of upper GI bleeding in our setting and compares our results with those from other centers in the world. The study is intended to bridge the existing knowledge gap on the management of this potentially life-threatening condition in our centre.

## Methods

### Study design and setting

This was a descriptive retrospective study of patients who underwent upper GI endoscopy for upper GI bleeding at the endoscopy unit of Bugando Medical Centre (BMC) between March 2010 and September 2011. Bugando Medical Centre is a consultant, tertiary care and teaching hospital for the Catholic University of Health and Allied Sciences- Bugando (CUHAS-Bugando) and has a bed capacity of 1000. It provides tertiary-level health care to the general population of approximately 13 millions people in Mwanza city and other regions in northwestern Tanzania. The endoscopy unit of Bugando Medical Centre was established in 2006. The unit is run by two well trained endoscopists who are also consultant physicians and operates only from Monday to Friday except during week end days (Saturday and Sunday) where it operates on emergency basis.

### Study subjects

Subjects of this study included all upper GI bleeding patients who had upper gastrointestinal tract endoscopy as part of their workup. Patients who had incomplete or missed basic information were excluded from the study. Data on demographic profile, aetiological spectrum, clinical presentation, endoscopic findings, treatment modalities, and clinical outcome was collected from endoscopy unit and from patient files at the Medical Records department. Information on demographic characteristics, endoscopic findings, medical and/or surgical management and clinical outcome (transfusion requirements, length of hospital stay, rebleeding rate, mortality) of all patients were recorded in a special preformed questionnaire. *Endoscopic evaluation* of the bleeding lesion in case of peptic ulcer was defined according to the FORREST Classification as following: FI – Active bleeding (FIa – arterial, spurting hemorrhage, FIb – oozing hemorrhage), FII – Stigmata of recent haemorrhage (FIIa – Visible vessel, FIIb – Adherent clot, FIIc –Dark base - haematin covered lesion, FIII –Lesions without active bleeding [18]. Patients who had variceal type of upper GI bleeding were classified endoscopically according to the severity of bleeding into four grades (i.e. grades I-IV) [19]. *Endoscopic diagnosis* was considered to be accurate, if stigmata of active or recent bleeding were present, independently of the nature of the bleeding lesion. *Normal examination* was defined by the absence of any endoscopic abnormality. *Shock* was defined as a systolic blood pressure below 90 mmHg. *Rebleeding* was defined as a new bleeding episode during the first 72 hours of hospitalization after the initial bleeding has stopped. *The Rockall risk scoring system*[4] using clinical criteria (age, co-morbidity, presence of shock) and endoscopy (diagnosis, stigmata of recent haemorrhage) was used to identify patients at risk of developing adverse outcomes after acute upper gastrointestinal haemorrhage.

### Statistical analysis

Statistical package for social sciences (SPSS, version 17.0; Chicago, IL, USA) program was used for data analysis. The mean ± standard deviation (SD), median and ranges were calculated for continuous variables whereas proportions and frequency tables were used to summarize categorical variables. Continuous variables were categorized. Chi-square (χ^2^) test were used to test for the significance of association between the independent (predictor) and dependent (outcome) variables in the categorical variables. The level of significance was considered as P < 0.05. Multivariate logistic regression analysis was used to determine predictor variables that predict the outcome.

### Ethical consideration

Ethical approval to conduct the study was sought from the CUHAS-Bugando/BMC joint institutional ethic review committee before the commencement of the study.

## Results

### Demographic characteristics

A total of 1292 patients had fibreoptic upper GI endoscopy during the study period and 252 out of these had upper GI bleeding. Of these 12 patients were excluded from the study due to incomplete or missed basic information. Thus, 240(18.6%) patients were studied. One hundred and sixty-three (67.9%) patients were males while 77 (32.1%) were females with a male to female ratio of 2.1:1. Their ages ranged from 11 to 85 years with a mean (± standard deviation) and median age of 39.8 ± 16.0 and 37 years respectively. The modal age group was 31–40 years (30.8%). Most of patients (60.0%) were aged 40 years and below. The majority of patients, 189 (78.8%) came from the rural areas located a considerable distance from Mwanza City and most of them, 177 (73.8%) had either primary or no formal education. The majority of patients, 140 (58.3%) were fishermen and peasants accounting for 78 (55.7%) and 62(44.3%) patients respectively. The remaining patients were either students (28, 11.7%), businessmen (22, 9.2%) or employed as public servants (50, 20.8%).

### Clinical characteristics of patients

The vast majority of the patients (193, 80.4%) presented with haematemesis alone. Malaena alone was reported in 22 (9.2%) patients. Twelve (5.0%) patients reported both haematemesis and malaena while 2 (0.8%) patients had haematochezia. A history of non-steroidal anti-inflammatory drugs (NSAIDs) ingestion prior to the onset of bleeding was recorded in 19(7.9%) patients and was significantly associated with the cause of the bleeding episode (P < 0.001). The use of alcohol and smoking was reported in 124(51.7%) and 92(38.3%) patients respectively. Alcohol use and smoking was not significantly associated with the etiology of the hemorrhagic episode (P > 0.001). Previous history of peptic ulcer disease was reported in 22(9.2%) of the patients. Sixty-five (27.1%) patients had underlying illness. Of these, 11 (16.9%) patients had chronic liver diseases, out of which four (36.4%) patients had clinical evidence of hepatic decompensation (e.g. ascites). Nine (13.8%) patients had portal hypertension, seven (10.8%) had hypertensive heart diseases, five (7.6%) had renal diseases and 3 (4.6%) had chronic chest infections. Diabetic mellitus, sickle cell diseases, asthma, congestive cardiac failure and malignancies were reported in six (9.2%) patients each respectively. HIV status was known in 214 (89.2%) patients. Of these, 9 (3.8%) patients were HIV positive and the remaining 205 (85.4%) patients were HIV negative. HIV status was not known in 26 (10.8%) patients. Hemorrhagic shock (systolic blood pressure < 90 mmHg) was reported in 68 (28.3%) patients.

### Endoscopic diagnosis

The source of bleeding was endoscopically identified in 232 (97.7%) patients and in eight (3.3%) patients no source of bleeding could be identified. Endoscopy was performed within 24 hours of the bleeding episode in 128 patients (53.3%). The mean time interval between the request to the endoscopic unit and the performance of the urgent endoscopy for in-patients with acute upper GI bleeding was 6.8 ± 1.2 hours. All endoscopic examinations were performed during working hours. Endoscopic examinations for outpatients were done on booking basis and the booking time ranged from 1 day to 34 days with a mean of 14.2± 6.4 days. An accurate diagnosis was established significantly more often in patients who presented with haematemesis (P = 0.015), and in patients who underwent endoscopy within 24 hours of the bleeding onset (P = 0.013).

Oesophageal varices were the most frequent cause of upper GI bleeding (51.3%) whereas peptic ulcers (i.e. duodenal ulcers (14.2%) and gastric ulcers (10.8%) were the most common causes of non-variceal bleeding accounting for 25.0% of cases. The endoscopic grades of patients, who had variceal type of upper GI bleeding were grade I 6(4.9%) patients, grade II 17 (13.8%), grade III 54 (43.9%), and grade IV 46 (37.4%) where as the endoscopic grades of patients, who had peptic ulcers were classified according to the Forrest classification as follows; grade Ia 2 (3.4%) patients, grade Ib 4 (6.9%), grade IIa, 2 (3.4%), grade IIb 9(15.5%), grade IIc 2 (3.4%) and grade III 39(67.2%). Erosive mucosal disease (oesophagitis, gastritis and duodenitis) accounted for 17.5% of cases with gastritis being the commonest representing 12.9% of all cases of upper GI bleeding. Among the patients who had history of non-steroidal anti-inflammatory drugs (NSAIDs) ingestion prior to the onset of bleeding, 14 (73.7%) had erosive mucosal disease. Histopathological examination of gastric ulcers revealed malignancies (adenocarcinoma) in 12 (46.2%) patients. The main bleeding lesions identified at upper GI endoscopy are shown in Table 1.

**Table 1:** Etiological spectrum and endoscopic findings of upper GI bleeding

**Aetiology/endoscopic findings**	**Frequency**	**Percentage**
Oesophageal varices	123	51.3
Duodenal ulcers	34	14.2
Gastritis	31	12.9
Gastric ulcers	26	10.8
Duodenitis	7	2.9
Oesophagitis	4	1.7
Oesophageal candidiasis	3	1.3
Gastric polyp	2	0.8
GERD	1	0.4
Oesophageal ulcers	1	0.4
Normal finding	8	3.3
Total	240	100

Key: GERD = Gastro-esophageal reflux disease.

### Admission and treatment patterns

A total of 144 (60.0%) patients were admitted for the management of upper GI bleeding. Of these, 12 (8.3%) patients were admitted to the intensive care unit (ICU) for ventilatory support. The remaining 96 (40.0%) patients were managed as an outpatient. The pattern of treatment is shown in Table 2.

i. Conservative (Medical) treatment

**Table 2:** Treatment pattern among patients with upper GI bleeding

**Treatment pattern**	**Number of patients**	**Percentage**
Medical (conservative) treatment	146	60.8
Endoscopic treatment	74	30.8
Surgical treatment	14	5.8
Not recorded	6	2.5
Total	240	100

The majority of patients (60.8%) were offered conservative medical treatment only, including intravenous fluid replacement with normal saline and Ringer’s lactate, intravenous proton pump inhibitors, antibiotics and blood transfusion as appropriate.

The majority of patients with hemodynamic instability were admitted to the intensive care unit.

ii. Endoscopic treatment

Endoscopic treatment was performed in 30.8% of patients and it was done only in patients who had esophageal varices. This included endoscopic variceal band ligation (EVBL) in 72 (97.3%) patients and the remaining 2 (2.7%) patients had endoscopic sclerotherapy. Most patients who underwent endoscopic treatment had either active bleeding or stigmata of recent bleeding at endoscopy. No patients with non-variceal bleeding had endoscopic treatment due to lack of facilities. Permanent hemostasis was achieved in 63(85.1%) of the patients at the first endoscopic intervention, and in 60% of the patients after rebleeding.

iii. Surgical treatment

Surgery was performed in only 5.8% of patients for upper GI bleeding. The indications and type of surgery performed are shown in table 3 and 4.

**Table 3:** Indications for surgery (N = 14)

**Indications for surgery**	**Frequency**	**Percent**
Bleeding duodenal ulcers not responding to either medical or endoscopic treatment	6	42.9
Gastric malignancy	5	35.7
Duodenal ulcers with gastric outlet obstruction	2	14.3
Bleeding duodenal ulcers associated with perforation	1	7.1

**Table 4:** Types of Surgery

**Type of surgery**	**Frequency**	**Percent**
Over-sewing or under-running of an ulcer	6	42.9
Gastro-jejunostomy	4	28.6
Gastrectomy	3	21.4
Graham’s omental patch (Graham’s omentopexy)	2	14.3

### Clinical outcome and rockall scoring system

The clinical outcome of patients was defined in terms of blood transfusion requirement, rebleeding, need for emergency surgery, length of hospital stay and mortality and it was assessed using the Rockall scoring system (Table 5). The Rockall’s score was calculated in 224 (93.3%) patients. Of these, 80 (35.7%) patients had Rockall score <3 (low risk group) whereas 110 (49.1%) and 34 (15.2%) patients had Rockall score 3–8 (medium risk group) and >8 (high risk group) respectively. Data for calculation of the Rockall score was missing in 16 (6.7%). The Rockall score ranged from 1–10 with a mean (±SD) of 3.8 (±1.6). Increased Rockall score was significantly associated with increased risk of rebleeding (P = 0.001), need for blood transfusion (P = 0.016), length of hospital stay (P = 0.011) and mortality (P = 0.000).

i. Blood transfusion requirement

**Table 5:** The Rockall scoring system

**Variables**	**Responses**	**Scores**
**Age (in years)**	< 60	0
	60-79	1
	>80	2
**Shock**	No shock	0
	Tachycardia (SBP > 100 mmHg, Pulse > 100 beats/minute)	1
	Hypotension (SBP < 100 mmHg, Pulse > 100 beats/minute)	2
**Co-morbidity**	None	0
	Cardiac failure, IHD, any major co-morbidity	2
	Renal/liver failure, metastatic malignancy	3
**Diagnosis (post-endoscopy)**	Mallory-Weiss tears	0
	All other diagnoses	1
	Malignancy of the upper GI tract	2
**Stigmata of recent haemorrhage**	None	0
	Blood, adherent clot, spurting vessel	2

Keys: SBP = systolic blood pressure, IHD = ischemic heart disease, GI = gastrointestinal.

Interpretation

Add up the criteria scores to get value.

< 3 indicates good prognosis (low risk for developing adverse outcomes).

3–8 indicates moderate prognosis (medium risk for developing adverse outcomes).

> 8 indicates poor prognosis (high risk for developing adverse outcomes).

One hundred thirty-two (55.0%) patients had hemoglobin less than 8 g/dl and 140 (58.3%) patients received blood transfusion for the upper GI bleeding. Transfusion requirements were 2.3 ± 1.4 units of packed red blood cells (RBCs) per patient. The need for blood transfusions was significantly higher in patients who underwent surgery compared to patients who had either medical or endoscopic treatment (P < 0.001). Transfusion requirement was significantly higher in non-survivors than in survivors (P = 0.021) and it was also strongly associated with increasing Rockall scores (P = 0.004).

ii. Rebleeding

Rebleeding was reported in 8 (3.3%) patients. of these, 5 (62.5%) had variceal bleeding and the remaining 3 (37.5%) patients had non-variceal bleeding. These differences were statistically significant (P = 0.032). The incidence of variceal re-bleeding was significantly higher in injection sclerotherapy treated group than in those treated with EVBL (23.7% versus 7.6%) (P = 0.001). The presence of a non-bleeding visible vessel in patients with peptic ulcer disease was significantly associated with rebleeding (P = 0.006). Rebleeding was also strongly associated with increasing Rockall scores (P = 0.023).

iii. Need for emergency surgery

Emergency surgery was required in 7(50.0%) patients. Of these, six (85.7%) patients underwent emergency surgery due to bleeding duodenal ulcers not responding to both medical and endoscopic treatment, and the remaining patient (14.3%) had emergency surgery due to bleeding duodenal ulcers associated with perforation. The need for emergency surgery was significantly associated with increasing risk of death (P = 0.015).

iv. Length of hospital stay

The overall length of hospital stay ranged from 1 day to 28 with a median of 8 days. The median length of hospital stay was significantly longer in patients who underwent surgical treatment (13 days) than in those who had either medical (4 days) or endoscopic treatment (6 days) (P = 0.045). The length of hospital stay also increased with higher Rockall scores (P = 0.002).

v. Mortality

The overall mortality rate was 11.7% (28 deaths). Patients who underwent surgical treatment (42.9%, 6/14) had significantly higher mortality than those who had either medical (7.5%, 11/146) or endoscopic treatment (13.5%, 10/74) (P < 0.001).). Mortality in patients with variceal bleeding (17.6%), hemorrhagic shock (21.3%), hepatic decompensation (23.5%), co-morbidities (35.3%), HIV infection (47.1%), elderly patients aged > 60 years (14.7%) and in patients with malignancy (24.8%) was significantly higher than in their counterparts (P < 0.001). Rebleeding was also associated with increasing risk of death (P = 0.012). Mortality was also strongly associated with increasing Rockall scores (P = 0.000).

## Discussion

Upper gastrointestinal bleeding is a common reason for emergency hospital admissions and a major cause of morbidity and mortality worldwide [1,20]. In this review, males constituted the larger proportion of cases in all age groups. The two-fold increase in the number of males with upper GI bleeding compared to females in the present study is similar to what was reported in other studies [8,13,15,20]. We could not establish the reason for the male predominance. The mean age of our patients was 39.8 years which is lower than the age reported in western studies [9,13,15,21]. This could just be a reflection of the generally older population of the west.

The majority of patients in this study were fishermen and peasants. Similar finding was reported by Elliott [22]. Fishermen and peasants in most developing countries like ours work in environment favourable for contracting diseases like schistosomiasis which lead to portal hypertension.

In agreement with other studies [21,23], the vast majority of the patients presented with haematemesis alone which is at variance with other reports which reported malaena as the most common presentation [17,24]. Other studies reported both haematemesis and malaena as the most common presentation [15,25]. These differences in clinical presentation reflect differences in the pattern and severity of the disease from one study to another. The clinical presentation of the bleeding episode in the present study was also found to be associated with the accuracy of the endoscopic diagnosis. Haematemesis is probably a most threatening event to patients, that may contribute to an earlier seek for medical attention.

The use of NSAIDS is a well known risk factor for upper gastrointestinal bleeding [24,26]. This fact is confirmed by our findings that 7.9% of all the patients had taken NSAIDS prior to the onset of bleeding. A history of non-steroidal anti-inflammatory drugs (NSAIDs) ingestion prior to the onset of bleeding was reported in 73.7% of patients who had erosive mucosal disease indicating that NSAIDS induce bleeding primarily through mucosal erosion. NSAIDs abuse especially indiscriminate use and purchase of these drugs across the counter has increased the risk of bleeding mainly from erosive mucosal disease and peptic ulcers. Regulations to discourage the dispensing and prescription of drugs by unqualified personnel are of paramount important in reducing this negative trend. Although the consumption of alcohol is a well-established risk factor for upper gastrointestinal bleeding [26], we could not find any significant association between alcohol abuse and specific bleeding lesions or a higher mortality rate.

Upper gastrointestinal (GI) bleeding has a broad differential diagnosis in a patient with HIV infection. Bleeding may result from conditions associated with HIV infection or may be completely unrelated [27]. The HIV seropositivity in our study was reported in 3.8% of cases which is lower than that in the general population [28]. The likelihood that the etiology of upper GI bleeding is related to HIV infection is primarily dependent on the patient’s CD4+ cell count; patients with lower CD4+ cell counts are more likely to have an HIV-related cause of the bleeding than are patients with higher CD4+ cell counts. However, due to the retrospective nature of our study, the CD4+ cell count results were missing in most HIV positive patients. Causes of upper GI bleeding in HIV-infected patients include Kaposi sarcoma, bacillary angiomatosis, mucosal ulcerations secondary to viral diseases (e.g., cytomegalovirus and herpes simplex virus infection), mycobacteria (e.g., Mycobacterium tuberculosis and MAC), (e.g., histoplasmosis), and non-Hodgkin lymphoma [27].

Upper GI endoscopy has been reported to be an effective initial diagnostic modality to localize the site and cause of bleeding in almost 85-90% of patients [29]. In the present study, the site of bleeding could be detected accurately in 97.7% of patients which is similar to that reported in other studies [12,15]. Mucosal lesions are well known to heal quickly and generally the time interval between the bleeding episode and the endoscopic procedure are known to influence the accuracy and the likelihood of finding a cause endoscopically [20]. Therefore, the availability of emergency endoscopy within 24 hours is most desirable. However, in real life situation, emergency endoscopy is rarely available in most health care centers in developing countries due to the insufficiency of well-trained endoscopists, teams or equipments. In the present study we found that the source of bleeding was established more often in those who underwent endoscopy within 24 hours of the bleeding episode than those who had it later.

Our study showed that esophageal varices were the commonest cause of upper GI bleeding which is in keeping with other studies conducted elsewhere[11,12,20]. This is contrary to the findings of most western studies where peptic ulcer disease has been identified as the commonest cause of upper GI bleeding [13-15]. This difference could be explained by the high prevalence of schistosomiasis in our set up which is a known risk factor for oesophageal varices. On the other hand, peptic ulcer disease which has been identified as the commonest cause of upper GI bleeding in the west [13-15] was the second commonest cause in our study with duodenal ulcer being more common than gastric ulcer. There is definite role for *Helicobacter pylori* infection in the etiopathogenesis of duodenal ulcer [30]. This finding could probably be due to the high prevalence of *H. pylori infection* in the population [31]. However, we could not determine the prevalence of the infection in this retrospective study, because tests for *H. pylori* status were not routinely performed in patients with acute upper GI bleeding during the period.

Erosive mucosal disease (oesophagitis, gastritis and duodenitis) ranked third at 17.5% which is in contrast to a previous study in Nigeria which reported erosive mucosal disease as the second commonest cause of upper GI bleeding [20].

In agreement with other studies [12,15,32], the majority of patients in the present study were treated non-surgically by either medical or endoscopic treatment. Surgery was performed in only 5.8% of patients for upper GI bleeding. Therapeutic endoscopy has recently become the primary modality employed in the management of upper gastrointestinal bleeding and over the past 20 years the need for urgent surgery has diminished and appears restricted to salvage-type procedures for the unstable exsanguinating patient or when endoscopic therapy combined with pharmacological intervention fails to secure permanent hemostasis [33]. Endoscopic therapy is a well-established procedure in the management of GI bleeding and can be used as an effective tool for selected patients [15,34]. Endoscopic therapy with either band ligation or injection sclerotherapy is an integral component of the management of acute variceal bleeding and of the long-term treatment of patients after a variceal bleed [34]. Overall, current data demonstrate clear advantages for using ligation in preference to sclerotherapy [35]. In the present study, the majority of patients (97.3%) with variceal bleeding were treated with endoscopic variceal band ligation (EVBL) with good results and only 2.7% of the patients had endoscopic sclerotherapy. All patients who underwent endoscopic sclerotherapy for variceal bleeding presented with re-bleeding requiring further EVBL. EVBL should therefore be considered the endoscopic treatment of choice in the treatment of esophageal varices. Low surgery rates in the present study implies that many patients who had upper GI bleeding received successful endoscopic therapy and/or effective medical therapy.

In agreement with other studies [15,35], the need for red blood cell transfusion in the present study was identified as a risk factor for death in upper GI bleeding. In this study, blood transfusion requirement increased with higher Rockall scores, probably implying that those with higher scores are more likely to be sicker with higher blood transfusion requirement.

Rebleeding rates have been reported in literature to occur in roughly 20-30% of patients and is associated with a high risk of death [36]. In the present study, re-bleeding was noted in 3.3%, a figure which is significantly low compared to that found in previous studies [15,36]. This could be attributed either to a comparable efficacy of endoscopic hemostasis, or to possible coincidental clinical characteristics of the different groups of patients. Further study is needed to confirm this observation. Rebleeding is associated with increased mortality; therefore, timely identification and aggressive management of patients at high risk for continued bleeding or rebleeding have become the major focus of upper GI bleeding therapy. Surgical consultation is recommended whenever rebleeding has been detected or in cases where patients have required more than 6 units of packed red blood cells.

The overall length of hospital stay in the present study was higher compared to that reported in previous studies [15,37]. In the present study, patients who underwent surgical treatment stayed longer in the hospital stay than those who had either medical or endoscopic treatment. Similar finding was also reported in other studies [15,36,37].This observation may be explained by the fact that patients undergoing surgical therapy are more likely to be sicker requiring long duration of hospital stay. The length of hospital stay also increased with higher Rockall scores, probably implying that those with higher scores are sicker, with more co-morbid disease or adverse outcomes.

The overall mortality rate in our patient population was 11.7% which is somewhat higher than the 10% reported in most western studies [7,17]. The high mortality rate in the present study could be due to delayed presentation to health facility and a pre-selection of high-risk patients with significant underlying medical conditions, to an academic and referral medical centre; but could also be partly compounded by comorbidities. Mortality rate was significantly higher in patients with variceal bleeding, shock, HIV infection, comorbidities, malignancy, age > 60 years and in patients with higher Rockall scores and those who underwent surgery.

As it occurs in other retrospective studies, loss of data is frequent and sometimes blunts the retrieval of fundamental information. Failure to document information or loss of information in the emergency room and medical wards, as well as loss of endoscopy records resulted in lack of recorded information in several areas and serves as an advice to the need for improving the quality and consistency of recording in different units.

## Conclusion

Oesophageal varices are the most common cause of upper gastrointestinal bleeding in our centre and responsible for the higher mortality in patients with upper GI bleeding. Fibreoptic endoscopy was able to identify the bleeding sites in most patients and diagnostic accuracy of fibreoptic endoscopy was related to the time interval between the onset of bleeding and endoscopy. It is therefore recommended that, early endoscopy should be performed, preferably within 24 hours of the onset of bleeding. Rockall score is feasible, accurate, effective system for predicting outcome in patients with upper GI bleeding and can be employed at our centre.

## Misc

Anthony Liwa, Rodrick Kabangila, Mariam Mirambo, Wolfgang Scheppach, Eliasa Mkongo, Mabula D. Mchembe and Phillipo L. Chalya are contributed equally.

## Competing interests

The authors declare that they have no competing interests. The study had no external funding. Operational costs were met by authors.

## Authors’ contributions

HJ and MK- study design, literature search, data analysis, manuscript writing & editing and did the endoscopy to all the patients. AL, RK, MM, WS, EM and MDM participated in data analysis, manuscript writing & editing whereas PLC participated in literature search, data analysis, manuscript writing & editing and submission of the manuscript. All the authors read and approved the final manuscript.
